# Complementary and alternative medicine use among outpatients during the 2015 MERS outbreak in South Korea: a cross-sectional study

**DOI:** 10.1186/s12906-020-02945-0

**Published:** 2020-05-13

**Authors:** Jung Hye Hwang, Hyun Jeong Cho, Hyea Bin Im, Young Sun Jung, Soo Jeung Choi, Dongwoon Han

**Affiliations:** 1 Department of Obstetrics and Gynecology, Hanyang University College of Medicine, Seoul, South Korea; 2 Department of Global Health and Development, Graduate School, Hanyang University, Seoul, South Korea; 3 Institute of Health Services Management, Hanyang University, Seoul, South Korea; 4 Graduate School of Public Policy, Hanyang University, Seoul, South Korea; 5 Department of Preventive Medicine, Hanyang University College of Medicine, 222 Wangsimni-ro, Seongdong-gu, Seoul, 04763 South Korea; 6 Seoul, South Korea

**Keywords:** Infectious disease, MERS, Community hospital, Complementary and alternative medicine, Korea

## Abstract

**Background:**

The 2015 MERS outbreak in South Korea was the largest event outside of the Middle East. Under such circumstances, individuals tend to resort to non-conventional solutions such as complementary and alternative medicine (CAM) to manage health. Thus, this study aims to examine characteristics of CAM use among outpatients in a community hospital setting during the 2015 MERS outbreak and to assess potential predictors of CAM use during the epidemic.

**Methods:**

A cross-sectional study was conducted among 331 patients (response rate: 82.75%) at a community hospital located in Seoul, South Korea. The survey instrument included 36 questions on the use of CAM, demographic characteristics, health status, and respondents’ perceptions and concerns about MERS infection. Chi-square test and logistic regression were conducted for data analysis using SPSS ver. 21.0., and a *p*-value of less than 0.05 was considered statistically significant for all analyses.

**Results:**

76.1% of respondents used one or more types of CAM modalities during the MERS outbreak. Consumption of easily accessible modalities such as multivitamin (51.2%) and food products (32.1%) was most popular, and the majority of CAM users relied on mass media (52.4%) and the internet (27.4%) to obtain information on CAM. The use of CAM was associated with age between 40 and 49, age over 50, prior CAM use, and dissatisfaction with the government response to the MERS outbreak.

**Conclusions:**

CAM was commonly used by outpatients during the 2015 MERS outbreak in Korea, and mass media was the main source of information. Establishing a media platform is of paramount importance to provide reliable information and ensure the safety of its use.

## Background

Recent emerging infectious diseases such as Severe Acute Respiratory Syndrome (SARS), Avian Influenza, and Middle East Respiratory Syndrome related coronavirus (MERS-CoV) continue to be a major public health concern [1]. The 2015 MERS outbreak in South Korea was the largest outbreak of MERS-CoV outside of the Middle East, resulting in 186 infected cases, 38 causalities, and 16,752 people being quarantined [2–4]. The characteristics and mechanisms of MERS transmission have been well investigated since the outbreak [5–7]. Specifically, MERS deaths in Korea can be attributed to the absence of effective vaccination and treatment for MERS, weak healthcare system for epidemic control, and ill-prepared hospitals with a deficiency in professional expertise, as well as negative pressure room for isolation of MERS patients [7–10]. Furthermore, increased incidences of nosocomial infection of MERS during the outbreak in 2015 elevated the fear, panic, and anger among the general public [11, 12].

As such, individuals exposed to risks and uncertainties tend to seek self-care measures and resort to non-conventional solutions such as complementary and alternative medicine (CAM) in order to demonstrate their autonomy, mitigate the risk, and make the situation more manageable [13–15]. Also, the usage is even greater among those with a chronic condition and report poorer health status [16–19]. Thus, the use of CAM among infectious diseases patients has gained popularity over the past decades, and a growing body of literature highlights its use against the threat of new and emerging infectious diseases such as HIV/AIDS, SARS, and H1N1 [20–26]. Furthermore, it is especially important to investigate the characteristics of CAM use in countries where the practice of traditional medicine is popular.

In Korea, the use of CAM is widespread and culturally embedded as traditional Korean medicine (TKM) is recognized as a part of mainstream medicine in the Korean healthcare system. TKM is easily accessible in communities as TKM providers are available in all three levels (primary, secondary, and tertiary) of healthcare facilities. Some of its modalities, such as acupuncture, moxibustion, cupping, and herbal preparations, have been covered by the national health insurance since 1987, and patients can choose between TKM and conventional medicine based on their preferences [27]. Therefore, practice and consumption of CAM modalities, including TKM, are common among Koreans to prevent and manage acute illnesses [28, 29].

However, previous studies on CAM use during epidemics focus on the clinical efficacy of CAM modalities [23–25]. Those provide limited information about the detailed pattern of CAM use, why specific CAM modalities were chosen, and where the patients obtained the information on CAM during the emerging infectious disease outbreak. Therefore, this study aims to examine patterns and motivations of CAM use among outpatients at a community hospital during the 2015 MERS outbreak in Korea and identify their source of information on non-conventional medicine use.

## Methods

### Study setting and participants

A descriptive cross-sectional study was conducted among patients attending the outpatient departments at a community hospital located in Seoul, South Korea. All outpatients over the age of 18 were eligible to participate in the survey. Exclusion criteria were those who were admitted to the inpatient unit at the time of the survey, unable to speak or understand the survey questionnaire.

### Study size

The formula for sample size determination using confidence interval (CI) of proportion was used to calculate the sample size: $n={z}_{\alpha /2}^2\bullet \frac{pq}{d^2}$, where n is the required sample size, p is the estimated proportion of CAM use among the general population (0.692) calculated from previous studies [30, 31], d is the margin of error set on 0.05, q is the proportion of people not suing CAM (1-p), and *z*_0.025_ is 1.96 which corresponds to the confidence interval of 95%. This equation gave the minimum sample size as 328, and we distributed a total of 400 survey questionnaires assuming a 20% non-response rate.

### Survey instrument

The semi-structured survey questionnaire was developed in the Korean language (See Additional file 1: Survey questionnaire). Because previous research tools on CAM use during emerging infectious disease outbreaks were unavailable, the survey instrument was developed based on earlier studies on CAM use [32–34]. In order to assess content and face validation of the survey instrument, the questionnaire was reviewed by three researchers to evaluate appropriateness, relevancy, clarity, adequacy, and organization of the questions. In addition, the questionnaire was pilot-tested on a sample of 20 participants to examine length, clarity, and difficulty of the questions, and a few items were revised based on the results.

The final version of the questionnaire included 36 items with both multiple-choice and open-ended questions, and respondents’ perceptions and concerns regarding the MERS outbreak were measured using a 5-point Likert scale. The survey was divided into four major sections: The first section included eight questions on health-related characteristics (smoking and drinking experience, the practice of regular exercise, and presence and perceived-severity of comorbidities) and the level of self-perceived interest in health-promoting behavior of respondents (1 = not at all, 5 = very much). The second section contained 11 questions regarding respondents’ perceived danger and severity of MERS outbreak (1 = not at all, 5 = very much), concerns about MERS infection (1 = not at all, 5 = very much), the opinions on the trust in government response during the MERS outbreak, and source of information about MERS outbreak. The third part consisted of seven questions on CAM use (CAM use prior to the outbreak, types of modalities used during the outbreak—the respondents who checked on any modalities were categorised as CAM users, source of information, the reason for CAM use, and intention to recommend CAM to others), and the last section had nine questions on respondents’ sociodemographic characteristics such as age, gender, marital status, level of education, occupation, level of income, religion, and area of residence.

### Data collection

The survey was conducted from November 26th to December 2nd, 2015. Two surveyors were trained and recruited for data collection, and the information about the research was given verbally to each respondent. The respondents were asked to complete the questionnaire by themselves, but if respondents did not understand certain questions, they were able to ask the surveyors for clarification. All participants were assured of their confidentiality and completed IRB-approved written informed consent before the survey. A total of 400 self-completed questionnaires were distributed, but 69 participants did not return or complete the questionnaire (response rate: 82.75%); therefore, the data of 331 respondents were included in the final analysis.

### Statistical methods

The collected data of 331 respondents were analyzed using the Statistical Package for Social Sciences (SPSS) version 21.0. Descriptive statistics were used to examine sociodemographic and health-related characteristics of respondents. Pearson’s Chi-square test was performed to identify differences in sociodemographic characteristics and perceptions and concerns experienced during the MERS outbreak between CAM users and non-users. Lastly, multivariate logistic regression analysis was conducted to determine potential predictors of CAM use during the MERS outbreak. The significant factors from previous chi-square test (gender, age, religion, marital status, occupation, smoking status, alcohol consumption, degree of concern about no treatment modality available for MERS, trust in the government response, the perceived danger of the MERS outbreak on one’s health, hand washing, and refraining from physical contact with others) were subjected to the regression analysis. A *p*-value of less than 0.05 was considered statistically significant for all analyses.

### Ethical clearance

The ethical approval of the study was obtained from the Institutional Review Board on Human Subjects Research and Ethics Committees, Hanyang University, Seoul, Korea (HYI-15-210-1).

## Results

### Sociodemographic characteristics of study participants

Table 1 presents the sociodemographic characteristics of respondents. The majority of respondents were women (68%), married (72.5%), received undergraduate education or higher (51.1%), have monthly household income higher than approximately 4000 USD (41.4%), and used CAM prior to the outbreak (54.7%).

**Table 1:** Sociodemographic characteristics of respondents

Characteristics	Total ***n*** = 331 (%)	CAM users ***n*** = 252 (%)	Non-users ***n*** = 79 (%)	***p***-value
**Gender**				
Male	106 (32.0)	70 (27.8)	36 (45.6)	0.003
Female	225 (68.0)	182 (72.2)	43 (54.4)	
**Age (years)**				
≤ 39	104 (31.4)	66 (26.2)	38 (48.1)	0.001
40–49	93 (28.1)	72 (28.6)	21 (26.6)	
≥ 50	134 (40.5),	114 (45.2)	20 (25.3)	
**Marital status**				
Single	91 (27.5)	62 (24.6)	29 (36.7)	0.035
Married	240 (72.5)	190 (75.4)	50 (63.3)	
**Level of education**				
Up to secondary school	162 (48.9)	125 (49.6)	37 (46.8)	0.668
Undergraduate or higher	169 (51.1)	127 (50.4)	42 (53.2)	
**Occupation**				
Professional	77 (23.3)	65 (25.8)	12 (15.2)	0.001
Office worker	80 (24.2)	48 (19.1)	32 (40.5)	
Self-employed	69 (20.8)	57 (22.6)	12 (15.2)	
Not employed	105 (31.7)	82 (32.5)	23 (29.1)	
**Monthly household income**				
Low (under 1 M won)	71 (21.5)	53 (21.0)	18 (22.8)	0.348
Middle (2 M ~ 4 M won)	123 (37.2)	99 (39.3)	24 (30.4)	
High (Over 4 M won)	137 (41.4)	100 (39.7)	37 (46.8)	
**Religion**				
No	136 (41.1)	93 (36.9)	43 (54.4)	0.006
Yes	195 (58.9)	159 (63.1)	36 (45.6)	
**Type of current illness**				
Chronic disease	140 (42.3)	113 (44.8)	27 (34.2)	0.094
Acute condition	191 (57.7)	139 (55.2)	52 (65.8)	
**Smoking status**				
No	237 (71.6)	191 (75.8)	46 (58.2)	0.003
Yes	94 (28.4)	61 (24.2)	33 (41.8)	
**Alcohol drinking**				
No	88 (26.6)	74 (29.4)	14 (17.7)	0.041
Yes	243 (73.4)	178 (70.6)	65 (82.3)	
**Prior CAM use**				
No	150 (45.3)	78 (31.0)	72 (91.1)	< 0.001
Yes	181 (54.7)	174 (69.0)	7(8.9)	

*CAM* Complementary and Alternative Medicine

76.1% of respondents have used at least one of the listed CAM modalities during the MERS outbreak, and significant differences in gender (*p* = 0.003), age (*p* = 0.001), marital status (*p* = 0.035), occupation (*p* = 0.001), religion (*p* = 0.006), smoking status (*p* = 0.003), alcohol consumption (*p* = 0.041), and prior use of CAM (*p* < 0.001) between CAM users and non-users were observed

### Types of CAM modalities used during the MERS outbreak

Types of CAM modalities used by respondents during the MERS outbreak are demonstrated in Table 2. A total of 252 CAM users were identified. Among natural products, the prevalence of multivitamin consumption was the highest (51.2%), followed by whole grain, brown rice, and black bean (32.1%), and probiotics (31.0%). The exercise was the most practiced modality among the mind and body practices (27.8%), and traditional Korean medicine was used by 6.3% of CAM users.

**Table 2:** Types of CAM modalities used during the MERS outbreak

CAM modalities	CAM users ***n*** = 252 (%)
**Natural product**^a^	
Multivitamin	129 (51.2)
Whole grain/Brown rice/Black Bean	81 (32.1)
Probiotics	78 (31.0)
Ginseng/Balloon Flower Root	75 (29.8)
Garlic/Ginger	55 (21.8)
Dietary supplements	54 (21.4)
Propolis	23 (9.1)
Cinnamon	12 (4.8)
Chinese yam	10 (4.0)
Green Vegetable Juice	3 (1.2)
**Mind and body Practices**^a^	
Exercise	70 (27.8)
Pray/Meditation	25 (9.9)
Massage	19 (7.5)
Yoga/Aerobic	9 (3.6)
Chiropractic	4 (1.6)
**Other complementary health approaches**^a^	
Traditional Korean medicine	16 (6.3)
Acupuncture	8 (3.2)

^a^ Columns do not add up to 100% due to use of multiple treatments

### Source of information on CAM

As presented in Table 3, the primary information source of CAM use was mass media (52.4%) and the internet (27.4%). The least popular source was pharmacists (2.8%).

**Table 3:** Source of information, Reason for CAM/non-CAM use, and intention to recommend

Variables	n (%)
**Source of information (*n*** **= 252)**^a^	
Mass media (Newspapers, Radio, TV)	132 (52.4)
Internet	69 (27.4)
Family or relatives	61 (24.2)
Friends or peers	50 (19.8)
Book or magazine	29 (11.5)
Others	15 (6.0)
Pharmacist	7 (2.8)
**Reasons for using CAM (*n*** **= 252)**^a^	
To improve the immune system	159 (63.1)
To prevent disease and maintain/promote health	134 (53.2)
To assist in the treatment of disease(s)	33 (13.1)
To aid in psychological comfort	20 (7.9)
To reduce pain	7 (2.8)
To minimize the adverse effects of conventional medicine	5 (2.0)
Others	2 (0.8)
**Intention to recommend (*n*** **= 252)**	
Yes	183 (72.6)
No	69 (27.4)
**Reason for not using CAM (*n*** **= 79)**	
Do not believe that CAM is relevant to disease treatment	22 (27.8)
Do not trust in the effectiveness of CAM	21 (26.6)
Others	16 (20.3)
Do not know about CAM	14 (17.7)
Worried about the adverse effects of CAM	6 (7.6)

^a^ Columns do not add up to 100% due to the selection of multiple answers

### Reason for CAM use and intention to recommend CAM

The most common reason for CAM use among 252 CAM users was to improve the immune system (63.1%), followed by to prevent disease and maintain/promote health (53.2%). Also, 72.6% of CAM users were willing to recommend CAM to others. As the reason for non-use of CAM among 79 non-CAM users, 27.8% responded that they do not believe that CAM is relevant to disease treatment, and 26.6% reported that they do not trust in the effectiveness of CAM (Table 3).

### Differences in perception, degree of concerns, and trust in the government response

Differences in perceptions, degree of concerns, and trust in government response between CAM users and non-users are demonstrated in Table 4. Majority of respondents obtained MERS-related information through mass media (58.6%), displayed a high degree of concern about contracting the disease (49.2%), not being able to visit the crowded area (47.1%), having insufficient information on MERS (42.3%), and unavailability of treatment modality for MERS infection (62.5%). However, only a few respondents rated their self-perceived likelihood of contracting the disease as high (24.2%). Hand-washing (78.5%) was the most practiced protective behavior against the MERS outbreak, followed by wearing a mask (56.2%), and refraining from going outside (44.7%).

**Table 4:** Difference in perception, concerns, and government trust between CAM users and non-users

Variables	Total ***n*** = 331 (%)	CAM users ***n*** = 252 (%)	Non-users ***n*** = 79 (%)	***p***-value
**Concern about own self and/or family members becoming infected**ª				
Low (1,2)	84 (25.4)	61 (24.2)	23 (29.1)	0.314
Moderate (3)	84 (25.4)	61 (24.2)	23 (29.1)	
High (4,5)	163 (49.2)	130 (51.6)	33 (41.8)	
**Concern about not being able to visit crowded area**ª				
Low (1,2)	79 (23.9)	57 (22.6)	22 (27.8)	0.054
Moderate (3)	96 (29.0)	67 (26.6)	29 (36.7)	
High (4,5)	156 (47.1)	128 (50.8)	28 (35.4)	
**Concern about not being able to visit health facilities**ª				
Low (1,2)	109 (32.9)	79 (31.3)	30 (38.0)	0.322
Moderate (3)	94 (28.4)	70 (27.8)	24 (30.4)	
High (4,5)	128 (38.7)	103 (40.9)	25 (31.6)	
**Concern about having insufficient information on MERS**ª				
Low (1,2)	98 (29.6)	75 (29.8)	23 (29.1)	0.207
Moderate (3)	93 (28.1)	65 (25.8)	28 (35.4)	
High (4,5)	140 (42.3)	112 (44.4)	28 (35.4)	
**Concern about no treatment modality available for MERS**ª				
Low (1,2)	51 (15.4)	33 (13.1)	18 (22.8)	0.017
Moderate (3)	73 (22.1)	51 (20.2)	22 (27.8)	
High (4,5)	207 (62.5)	168 (66.7)	39 (49.4)	
**Perceived likelihood of infection**°				
Low (1,2)	140 (42.3)	103 (40.9)	37 (46.8)	0.458
Moderate (3)	111 (33.5)	89 (35.3)	22 (27.8)	
High (4,5)	80 (24.2)	60 (23.8)	20 (25.3)	
**Perceived danger of MERS outbreak on health**^1^				
Low (1,2)	29 (8.8)	22 (8.7)	7 (8.9)	0.017
Moderate (3)	124 (37.5)	84 (33.3)	40 (50.6)	
High (4,5)	178 (53.8)	146 (57.9)	32 (40.5)	
**Perceived preventability of MERS infection**				
Possible	257 (77.6)	197 (78.2)	60 (75.9)	0.679
Not possible	74 (22.4)	55 (21.8)	19 (24.1)	
**Trust in the government response**				
Appropriate	133 (40.2)	100 (39.7)	33 (41.8)	0.020
Not appropriate	151 (45.6)	123 (48.8)	28 (35.4)	
No opinion	47 (14.2)	29 (11.5)	18 (22.8)	
Protective behaviors*				
Wearing a mask	186 (56.2)	144 (57.1)	42 (53.2)	0.534
Hand-washing	260 (78.5)	206 (81.7)	54 (68.4)	0.011
Refraining from going outside	148 (44.7)	117 (46.4)	31 (39.2)	0.262
Refraining from physical contact with other people	62 (18.7)	55 (21.8)	7 (8.9)	0.010
Resting	73 (22.1)	58 (23.0)	15 (19.0)	0.451
Visiting health facilities for consultation	10 (3.0)	7 (2.8)	3 (3.8)	0.644

ª Participants were asked to rate their degree of concern (1 = Not at all, 5 = Very much)

° Participants were asked to rate their self-perceived likelihood of contracting the disease (1 = Not at all, 5 = Very likely)

^1^ Participants were asked to rate their self-perceived danger of the MERS on health (1 = Not at all, 5 = Very much)

* Columns do not add up to 100% due to the selection of multiple answers

CAM users’ concern for the lack of definitive treatment for MERS infection (*p* = 0.017) and self-perceived danger of the MERS outbreak (*p* = 0.017) was significantly greater than that of non-CAM users. A more significant proportion of CAM users (48.8%) regarded government response to the MERS outbreak as inappropriate (*p* = 0.020), and CAM users practiced more hand-washing (*p* = 0.011) and refrained from physical contact with others (*p* = 0.010) compared to the non-users.

### Potential predictors of CAM use during the MERS outbreak

The result of multivariate logistic regression analysis is shown in Table 5. Age between 40 and 49 (OR: 2.753, CI: 1.121–6.761), age above 50 (OR: 3.574, CI: 1.290–9.905), CAM use prior to the outbreak (OR: 20.450, CI: 8.335–50.176), dissatisfaction with the government response to the outbreak (OR: 2.547, CI: 1.189–5.458) were associated with CAM use during the MERS epidemic.

**Table 5:** Multivariate logistic regression analysis for determining factors affecting CAM use during MERS

Variables	OR	95% CI	***p***-value
**Gender**			
Male	1	Ref	
Female	1.328	0.506–3.486	0.564
**Age (years)**			
≤ 39	1	Ref	
40–49	2.753	1.121–6.761	0.027
≥ 50	3.574	1.290–9.905	0.014
**Religion**			
No	1	Ref	
Yes	1.387	0.678–2.837	0.370
**Marital status**			
Married	1	Ref	
Single	1.321	0.546–3.199	0.537
**Occupation**			
Not employed	1	Ref	
Self-employed	2.275	0.806–6.423	0.121
Office worker	0.763	0.283–2.054	0.592
Professional	1.274	0.459–3.533	0.642
**Smoking status**			
No	1	Ref	
Yes	0.610	0.240–1.553	0.300
**Alcohol drinking**			
No	1	Ref	
Yes	1.389	0.566–3.406	0.473
**Prior CAM use**			
No	1	Ref	
Yes	20.450	8.335–50.176	< 0.001
**Concern about no treatment modality available for MERS**ª			
Low	1	Ref	
Moderate	1.204	0.425–3.407	0.727
High	1.625	0.612–4.310	0.330
**Trust in the government response**			
Appropriate	1	Ref	
Inappropriate	2.547	1.189–5.458	0.016
No opinion	1.170	0.420–3.263	0.764
**Perceived danger of MERS outbreak on health**^b^			
Low	1	Ref	
Moderate	0.850	0.228–2.844	0.736
High	1.066	0.298–3.816	0.922
**Frequent hand washing during the outbreak**			
No	1	Ref	
Yes	2.017	0.922–4.413	0.079
**Refraining from physical contact with others**			
No	1	Ref	
Yes	2.029	0.727–5.659	0.176

ª Participants were asked to rate their degree of concern (1 = Not at all, 5 = Very much)

^b^ Participants were asked to rate their self-perceived danger of the MERS on health (1 = Not at all, 5 = Very much)

## Discussion

This research is the first attempt to understand the prevalence and determinants of CAM use among patients during the MERS outbreak in Seoul, South Korea. While the efficacy of many CAM therapies has yet to be demonstrated, previous studies reported that CAM was commonly used during the outbreak of infectious diseases, such as malaria, dengue, H1N1, SARS, and the common cold to improve health [21–23, 25, 32, 35, 36]. Our findings also revealed a high utilization rate of CAM (76.1%) during the MERS outbreak, yet similar prevalence was reported during a non-epidemic period in Korea, showing an overall rise in CAM use among Koreans [30, 37, 38]. Furthermore, the prevalence of CAM use during the MERS outbreak was similar to that of cancer patients [39, 40], yet higher than that of chronic disease patients in Korea [41–43].

To date, the reasons and motivations for seeking CAM during epidemics vary across studies [14, 21, 22, 32]. However, during the MERS outbreak in Korea, the leading motivations for trying CAM were to improve the immune system and to prevent diseases. During the previous outbreaks of severe influenza, it was also documented that CAM was used for direct treatment against infectious diseases in various countries [22–24, 35, 44]. This implies that during infectious disease outbreaks, CAM is used not only to prevent the infection through the achievement of overall well-being and enhancement of immune function, but also to cure the disease directly by combating against the virus.

As there are multiple reasons behind the utilization of CAM, the use of CAM products differed among studies. In Korea, consumption of natural products such as a multivitamin, whole grain, and probiotics was popular during the MERS outbreak, which were the modalities that are widely used for the management and treatment of common cold and influenza-like illnesses [24]. Such similarities in modalities used support existing findings that people’s decision to use particular CAM is influenced by specific physical symptoms experienced and self-assessed health care needs [45, 46]. Our findings also show differences in the use of self-administered modalities (multivitamin, nutritional supplements, food products, and exercise) and practitioner-administered modalities (massage, yoga, and traditional Korean medicine). Higher preference of self-administered CAM modalities in our study implies that in order to protect themselves from an outbreak event, people are more likely to rely on self-medication and minimize the risk of exposure. In fact, a high proportion of Koreans during the 2015 MERS outbreak avoided the crowded area and health facilities to prevent infection [2].

Differences in the method of obtaining CAM information were also observed. During the MERS outbreak in Korea, the most common sources were mass media (newspaper, television, radio) and the internet, which were the least popular among patients with chronic disease and non-airborne infectious disease [32, 47–49]. Observed dependence on the mass media can be attributed to many health professionals using the mass media to provide advice on precautionary and self-care measures during the outbreak of MERS. Furthermore, similar to the SARS epidemic in Hong Kong [50], the majority of respondents also reported the mass media as the main source of disease-related information during the MERS outbreak in Korea. These findings reflect the importance of mass media accessed by the public during health emergencies, as the information provided has a critical role in the formation of risk perception and influence health behavior [51–53].

Lastly, several factors were identified as potential predictors of CAM use during the MERS outbreak. As for sociodemographic characteristics, previous studies found female gender, younger age, and tertiary education level to be associated with CAM use among people with risk of disease development [54, 55]. However, on the contrary, only the higher age groups (being 40 to 49 years of age, being over 50 years of age) were more likely to use CAM in our study. This incongruency may be due to differences in the sample size of the study and characteristics of diseases, as in the case of infectious disease outbreaks, higher perceived vulnerability among elders could influence them to engage in health-promoting behaviors [14, 56]. In the case of health belief-related factors, those who used CAM prior to the outbreak and showed dissatisfaction with the government response to the outbreak were more likely to use CAM in the present study. Similarly, previous studies found distrust in the government and dissatisfaction with the conventional health care system to be associated with CAM use [16, 57]. These results suggest that individuals who find the formal health care system to be unreliable resort to CAM to compensate for the lack of perceived-health care quality, to practice autonomy, and to protect themselves from health threats [16, 57–59]. However, the findings remain inconclusive, and further research is needed to improve our understanding of the determinants of CAM use during epidemic events.

Limitations of this study include recall bias and lack of variability in research locations. The survey was implemented in November and December of 2015, which was near the end of the MERS epidemic in Korea, so recall bias may affect the result. Also, the participants of the study were selected from patients visiting outpatient departments of a community hospital in Seoul; therefore, the result of the study may not fully represent those of other people in Korea. Lastly, cross-sectional studies can only infer the association between CAM use and potential predictor variables.

## Conclusion

During the 2015 MERS outbreak in Korea, the prevalence of CAM use was considerably high among patients with older age, prior CAM use, and dissatisfaction with the government response to the MERS outbreak. Also, the majority of respondents reported the mass media and the internet as the main source of CAM and disease-related information. In the era where emerging infectious diseases continue to be a major public health concern, the effective use of mass media and information and communication technology should be considered to achieve efficient delivery of health information and to promote behavior changes in situations of vulnerability. Therefore, public health authorities should build an integrated platform that provides proven and reliable information during public health emergencies to protect the health of citizens.

## Supplementary information

**Additional file 1.** Survey questionnaire. Survey questionnaire developed to examine the pattern of CAM use among Koreans during the 2015 MERS outbreak in Korea.

## Data Availability

The data will be accessible by contacting the corresponding author of this study.

## Abbreviations

CAM: complementary and alternative medicine
H1N1: Influenza A virus subtype H1N1
MERS: Middle East respiratory syndrome
OPD: Outpatient department
SARS: Severe Acute Respiratory Syndrome

## References

[1] Jones KE, Patel NG, Levy MA, Storeygard A, Balk D, Gittleman JL, Daszak P. Global trends in emerging infectious diseases. Nature. 2008;451(7181):990–3. 10.1038/nature06536 PMC5960580 18288193

[2] Lee SY, Yang HJ, Kim G, Cheong H-K, Choi BY. Preventive behaviors by the level of perceived infection sensitivity during the Korea outbreak of Middle East Respiratory Syndrome in 2015. Epidemiol Health. 2016;38. 10.4178/epih.e2016051 PMC5309729 28092927

[3] Kim K, Tandi T, Choi J, Moon J, Kim M (2017). Middle East respiratory syndrome coronavirus (MERS-CoV) outbreak in South Korea, 2015: epidemiology, characteristics and public health implications. J Hosp Infect.

[4] Fu C, Wang S (2016). Nosocomial infection control in healthcare settings: protection against emerging infectious diseases. Infectious Diseases Poverty.

[5] Park J-E, Jung S, Kim A, Park J-E (2018). MERS transmission and risk factors: a systematic review. BMC Public Health.

[6] Nam H-S, Park JW, Ki M, Yeon M-Y, Kim J, Kim SW (2017). High fatality rates and associated factors in two hospital outbreaks of MERS in Daejeon, the Republic of Korea. Int J Infect Dis.

[7] Ki M (2015). 2015 MERS outbreak in Korea: hospital-to-hospital transmission. Epidemiol Health.

[8] Choi JW, Kim KH, Cho YM, Kim SH. Current epidemiological situation of Middle East respiratory syndrome coronavirus clusters and implications for public health response in South Korea. J Korean Med Assoc. 2015;58(6):487–97.

[9] Na BJ, Kim D-H (2015). Improving capability of local public hospital andhealth center against newly emerging infectiousdiseases after Middle East respiratory syndromeepidemic in Korea. J Korean Med Assoc.

[10] Park C-B: Functions and roles of public healthcare for controlling infectious diseases. J Korean Med Assoc/Taehan Uisa Hyophoe Chi 2015, 58(7)..

[11] Park C-B. Functions and roles of public healthcare forcontrolling infectious diseases. J Korean Med Assoc. 2015;58(7):617–23.

[12] Oh M-D, Park WB, Park S-W, Choe PG, Bang JH, Song K-H, Kim ES, Kim HB, Kim NJ: Middle East respiratory syndrome: what we learned from the 2015 outbreak in the Republic of Korea. Korean J Internal Med 2018, 33(2):233. 10.3904/kjim.2018.031 PMC5840604 29506344

[13] Mitchell M, McClean S (2013). Pregnancy, risk perception and use of complementary and alternative medicine. Health Risk Soc.

[14] Siu JYM, Sung HC, Lee W (2007). Qigong practice among chronically ill patients during the SARS outbreak. J Clin Nurs.

[15] Omar UH, Putit L (2012). Consumer behavioral intention to use complementary alternative medicine. Identity.

[16] Ventola CL (2010). Current issues regarding complementary and alternative medicine (CAM) in the United States: part 1: the widespread use of CAM and the need for better-informed health care professionals to provide patient counseling. Pharmacy Therapeutics.

[17] Harris P, Cooper K, Relton C, Thomas K (2012). Prevalence of complementary and alternative medicine (CAM) use by the general population: a systematic review and update. Int J Clin Pract.

[18] Metcalfe A, Williams J, McChesney J, Patten SB, Jetté N (2010). Use of complementary and alternative medicine by those with a chronic disease and the general population-results of a national population based survey. BMC Complement Altern Med.

[19] Upchurch DM, Chyu L, Greendale GA, Utts J, Bair YA, Zhang G, Gold EB (2007). Complementary and alternative medicine use among American women: findings from the National Health Interview Survey, 2002. J Women's Health.

[20] Ha K-C, Kim M-G, Oh M-R, Choi E-K, Back H-I, Kim S-Y, Park E-O, Kwon D-Y, Yang H-J, Kim M-J (2012). A placebo-controlled trial of Korean red ginseng extract for preventing influenza-like illness in healthy adults. BMC Complement Altern Med.

[21] Hollenberg D, Zakus D, Cook T, Xu X (2008). Re-positioning the role of traditional, complementary and alternative medicine as essential health knowledge in global health: Do they still have a role to play?. World Health Population.

[22] Ganjhu RK, Mudgal PP, Maity H, Dowarha D, Devadiga S, Nag S, Arunkumar G (2015). Herbal plants and plant preparations as remedial approach for viral diseases. Virusdisease.

[23] Leung P-C (2007). The efficacy of Chinese medicine for SARS: a review of Chinese publications after the crisis. Am J Chinese Med.

[24] Mousa HA (2017). Prevention and treatment of influenza, influenza-like illness, and common cold by herbal, complementary, and natural therapies. J Evid Based Complementary Altern Med.

[25] Liu X, Zhang M, He L, Li Y. Chinese herbs combined with Western medicine for severe acute respiratory syndrome (SARS). Cochrane Database Syst Rev. 2012;10. 10.1002/14651858.CD004882.pub3 PMC6993561 23076910

[26] Syed IA, Sulaiman SAS, Hassali MA, Thiruchelvam K, Syed SH, Lee CK (2016). Beliefs and practices of complementary and alternative medicine (CAM) among HIV/AIDS patients: a qualitative exploration. Eur J Integrative Medicine.

[27] Choi JH, Kang SW, You CH. The determinants of choosing traditional Korean medicine or conventional medicine: findings from the Korea HealthPanel. Evid. Based Complement Alternat Med. 2015;2015. Article ID 147408:9. 10.1155/2015/147408. 10.1155/2015/147408 PMC4496497 26199631

[28] Kim H, Song M-J (2012). Traditional plant-based therapies for respiratory diseases found in north Jeolla Province, Korea. J Altern Complement Med.

[29] Jo J, Lee SH, Lee JM, Lee H, Kwack SJ, Kim DI (2016). Use and safety of Korean herbal medicine during pregnancy: a Korean medicine literature review. Eur J Integrative Med.

[30] Ock SM, Choi JY, Cha YS, Lee J, Chun MS, Huh CH, Lee SY, Lee SJ (2009). The use of complementary and alternative medicine in a general population in South Korea: results from a national survey in 2006. J Korean Med Sci.

[31] Park H-S, Cho G-Y, Kim M-O, Lee S-R (2005). A study on use of complementary-alternative therapy in middle-aged women. Korean J Women Health Nurs.

[32] Ching S, Ramachandran V, Gew LT, Lim SMS, Sulaiman WAW, Foo YL, Zakaria ZA, Samsudin NH, Lau PCMC, Veettil SK (2015). Complementary alternative medicine use among patients with dengue fever in the hospital setting: a cross-sectional study in Malaysia. BMC Complement Altern Med.

[33] Hwang JH, Han DW, Yoo EK, Kim W-Y (2014). The utilisation of complementary and alternative medicine (CAM) among ethnic minorities in South Korea. BMC Complement Altern Med.

[34] Hwang JH, Kim W-Y, Ahmed M, Choi S, Kim J, Han DW. The use of complementary and alternative medicine by Korean breast cancer women: is it associated with severity of symptoms? Evid Based Complement Alternat Med. 2015;2015(Article ID 182475):7. 10.1155/2015/182475. 10.1155/2015/182475 PMC4681796 26770251

[35] Arora R, Chawla R, Marwah R, Arora P, Sharma R, Kaushik V, Goel R, Kaur A, Silambarasan M, Tripathi R. Potential of complementary and alternative medicine in preventive management of novel H1N1 flu (swine flu) pandemic: thwarting potential disasters in the bud. Evid Based Complement Alternat Med. 2011;2011. 10.1155/2011/586506 PMC2957173 20976081

[36] Nahas R, Balla A (2011). Complementary and alternative medicine for prevention and treatment of the common cold. Can Fam Physician.

[37] Lee SI, Khang YH, Lee MS, Koo HJ, Kang W, Hong CD (1999). Complementary and alternative medicine use in Korea: prevalence, pattern of use, and out-of-pocket expenditures. Korean J Preventive Med.

[38] Lim B, Min J, Jang U, Min M (2004). The use and expenditure of the complementary and alternative medicine in Korea. J Korean Oriental Med Society.

[39] Kim MJ, Lee SD, Kim DR, Kong YH, Sohn WS, Ki SS, Kim J, Kim YC, Han CJ, Lee JO (2004). Use of complementary and alternative medicine among Korean cancer patients. Korean J Internal Med.

[40] Chang SB, Lee TW, Kim S, Yoo IY, Kim IS, Kang KH, Lee MK, Jang YH (2006). A study of complementary and alternative medicine used by cancer patients in Korea. J Korean Acad Adult Nurs.

[41] Hwang BY, Park MN, Choi HS, Choi CW, Yoo JH, Kang HM, Park MJ (2006). The current status of complementary-alternative medicine for asthmatics in Korea: experience in one tertiary care hospital. Tuberculosis Respiratory Diseases.

[42] Lee MS, Lee MS, Lim HJ, Moon SR (2004). Survey of the use of complementary and alternative medicine among Korean diabetes mellitus patients. Pharmacoepidemiol Drug Saf.

[43] Shin Y-I, Yang C-Y, Joo M-C, Lee S-G, Kim J-H, Lee MS (2008). Patterns of using complementary and alternative medicine by stroke patients at two university hospitals in Korea. Evid Based Complement Alternat Med.

[44] Tao ZG, Yang YX, Shi WN, Xue MM, Yang WQ, Song ZJ, Yao CL, Yin J, Shi DW, Zhang YP (2013). Complementary and alternative medicine is expected to make greater contribution in controlling the prevalence of influenza. Bioscience Trends.

[45] Robinson A, Chesters J, Cooper S (2007). People's choice: complementary and alternative medicine modalities. Complement Health Pract Rev.

[46] Tsao JC, Dobalian A, Myers CD, Zeltzer LK (2005). Pain and use of complementary and alternative medicine in a national sample of persons living with HIV. J Pain Symptom Manag.

[47] Busari AA, Mufutau MA (2017). High prevalence of complementary and alternative medicine use among patients with sickle cell disease in a tertiary hospital in Lagos, south west, Nigeria. BMC Complement Altern Med.

[48] Hu H, Li G, Duan J, Arao T. Prevalence, Purposes, and Perceived Effectiveness of Complementary and Alternative Medicine Use in a Hypertension Population: A Questionnaire Survey. ISRN Public Health. 2013;2013:137472.

[49] Kucukoner M, Bilge Z, Isıkdogan A, Ali Kaplan M, Inal A, Urakci Z. Complementary and alternative medicine usage in Cancer patients insoutheast of Turkey. Afr Tradis Compl Alternat Med. 2012;10(1):21–25. 10.4314/ajtcam.v10i1.4 PMC3746353 24082321

[50] Lau J, Yang X, Tsui H, Kim J (2003). Monitoring community responses to the SARS epidemic in Hong Kong: from day 10 to day 62. J Epidemiol Community Health.

[51] Kim J, Jung M (2017). Associations between media use and health information-seeking behavior on vaccinations in South Korea. BMC Public Health.

[52] Seo M (2016). Effects of risk information seeking and processing on MERS preventive behaviors and moderating roles of SNS use during 2015 MERS outbreak in Korea. Korean J Communication Information.

[53] Advani R, Naess H, Kurz M (2016). Mass media intervention in Western Norway aimed at improving public recognition of stroke, emergency response, and acute treatment. J Stroke Cerebrovasc Dis.

[54] Field K, Jenkins M, Friedlander M, McKinley J, Price M, Weideman P, Keogh L, McLachlan S, Lindeman G, Hopper J (2009). Predictors of the use of complementary and alternative medicine (CAM) by women at high risk for breast cancer. Eur J Cancer.

[55] Kristoffersen AE, Sirois FM, Stub T, Hansen AH (2017). Prevalence and predictors of complementary and alternative medicine use among people with coronary heart disease or at risk for this in the sixth Tromsø study: a comparative analysis using protection motivation theory. BMC Complement Altern Med.

[56] de Zwart O, Veldhuijzen IK, Richardus JH, Brug J (2010). Monitoring of risk perceptions and correlates of precautionary behaviour related to human avian influenza during 2006-2007 in the Netherlands: results of seven consecutive surveys. BMC Infect Dis.

[57] Lee C, Whetten K, Omer S, Pan W, Salmon D (2016). Hurdles to herd immunity: distrust of government and vaccine refusal in the US, 2002–2003. Vaccine.

[58] Guillaud A, Allenet B, Pinsault N (2020). Does dissatisfaction with physicians lead patients to alternative practitioners?. Complement Ther Clin Pract.

[59] Kharroubi S, Chehab RF, El-Baba C, Alameddine M, Naja F. Understanding CAM use in Lebanon: findings from a National Survey. Evid Based Complement Alternat Med. 2018;2018. 10.1155/2018/4169159 PMC6083547 30147730

