# Supplementary material for: Complementary and alternative medicine use among outpatients during the 2015 MERS outbreak in South Korea: a cross-sectional study
Source: BMC Complement Med Ther. 2020 May 13;20:147. doi: 10.1186/s12906-020-02945-0 (PMC7220580; doi:10.1186/s12906-020-02945-0)
Supplement: Supplementary file 1 — Additional file 1. Survey questionnaire. Survey questionnaire developed to examine the pattern of CAM use among Koreans during the 2015 MERS outbreak in Korea. [file 12906_2020_2945_MOESM1_ESM.docx]

### Questions on current health status

1. How would you rate your current health status?

| Very poor | Poor | Neutral | Good | Very good |
| --- | --- | --- | --- | --- |
| ① | ② | ③ | ④ | ⑤ |

1. How would you rate your level of interest in health management?

| Very low | Low | Neutral | High | Very high |
| --- | --- | --- | --- | --- |
| ① | ② | ③ | ④ | ⑤ |

1. How often do you smoke cigarettes?

| Never | Currently smoking | Not anymore |
| --- | --- | --- |
| ① | ② | ③ |

1. How often do you consume alcoholic drinks?

| Never | 1 ~ 2 times a month | 1 ~ 2 times a week | ≥ 3-4 times a week |
| --- | --- | --- | --- |
| ① | ② | ③ | ④ |

1. Do you exercise regularly?

① Yes ② No

1. Are you currently undergoing treatment for any of the following chronic diseases? (“√” all that apply)

① High blood pressure

② Diabetes

③ Cerebrovascular disease (stroke, cerebral hemorrhage, etc.)

④ Heart disease (angina, myocardial infarction, etc.)

⑤ Liver disease (hepatitis, cirrhosis, etc.)

⑥ Cancer

⑦ Lung disease / Chronic lower respiratory disease (bronchitis, emphysema, etc.)

⑧ Joint diseases (back pain, knee, shoulder, neck, etc.)

⑨ Others (Please specify __________________)

⑩ None

1. How long did you have a chronic condition? (Based on primary condition)

| Less than 3 months | 3~6 months | 6 months-1 year | More than 1 year |
| --- | --- | --- | --- |
| ① | ② | ③ | ④ |

1. What is the level of perceived-severity of your current condition?

| Not at all | Minimal | Moderate | Severe | Very severe |
| --- | --- | --- | --- | --- |
| ① | ② | ③ | ④ | ⑤ |

**II. Questions on the perception of MERS**

- Middle East Respiratory Syndrome (MERS): Severe respiratory illness caused by coronavirus infection

1. How would you rate your level of concern on the following items during the MERS outbreak?

| Item | | Very  low | Low | Moderate | High | Very high |
| --- | --- | --- | --- | --- | --- | --- |
| 1. | Concern about own self and/or family members becoming infected | ① | ② | ③ | ④ | ⑤ |
| 2. | Concern about not being able to visit the crowded area | ① | ② | ③ | ④ | ⑤ |
| 3. | Concern about not being able to visit health facilities | ① | ② | ③ | ④ | ⑤ |
| 4. | Concern about lack of information on MERS | ① | ② | ③ | ④ | ⑤ |
| 5. | Concern about the lack of treatment modality available for MERS | ① | ② | ③ | ④ | ⑤ |

1. How would you rate your perceived-severity of the MERS outbreak when you heard about MERS mortality on the media?

| Not at all | Minimal | Moderate | Severe | Very severe |
| --- | --- | --- | --- | --- |
| ① | ② | ③ | ④ | ⑤ |

1. Do you think there is a high chance of contracting MERS, given your current health status?

| Very low | Low | Neutral | High | Very high |
| --- | --- | --- | --- | --- |
| ① | ② | ③ | ④ | ⑤ |

1. Do you feel that you have enough information on MERS?

| Not at all | Insufficient | Moderate | Sufficient | Very sufficient |
| --- | --- | --- | --- | --- |
| ① | ② | ③ | ④ | ⑤ |

1. Where did you get information on MERS? (“√” all that apply)

| ① Family / Relatives | ② Friends and peers |
| --- | --- |
| ③ Media (newspaper, radio, TV) | ④ Internet |
| ⑤ Books or magazines | ⑥ Pharmacist |
| ⑦ Others (__________________) |  |

1. What was your primary interest during the outbreak? (Select one)

① Current status on MERS outbreak (number of infected persons)

② MERS treatment modality (development of curative treatment and vaccines)

③ Personal hygiene (handwashing, wearing a mask, etc.)

④ Promoting personal immunity (folk remedies and activities to increase immunity)

⑤ Number of deaths due to MERS

⑥ Others (_______________________)

1. Do you think that following government response (Mayor's announcement) was appropriate during the MERS outbreak?

- The 35^th^ confirmed patient is a physician “A” who had contacted the 14^th^ patient. The physician “A” started to have mild symptoms from May 29^th^, the symptoms exacerbated on May 30^th^, and the physician “A” was finally MERS confirmed on June 1^st^. However, the physician “A” had attended a public event on May 30^th^ with 1,565 attendees.
- In response to the 35^th^ confirmed patient, Seoul Metropolitan Government has obtained a list of 1,565 attendees of the public event that the physician “A” had attended and plan to contact all attendees individually today to request for voluntary isolation.

① Appropriate ② Not appropriate ③ No opinion

1. How would you rate the perceived danger of the MERS outbreak on health?

| Not at all | Mild | Moderate | Dangerous | Very dangerous |
| --- | --- | --- | --- | --- |
| ① | ② | ③ | ④ | ⑤ |

1. Please check all MERS-related symptoms you have experienced during the MERS outbreak (“√” all that apply)

| ① Runny or stuffy nose | ② Sore throat |
| --- | --- |
| ③ Cough | ④ Fever |
| ⑤ Diarrhea | ⑥ Abdominal pain |
| ⑦ Difficulty breathing | ⑧ Rapid shallow breathing |
| ⑨ No symptoms | ⑩ Others (_______________________) |

1. Did you practice any of the following self-protective behaviors during the MERS outbreak? (“√” all that apply)

| ① Wear a mask | ② Handwashing |
| --- | --- |
| ③ Refrain from going out | ④ Refrain from physical contact with others |
| ⑤ Adequate rest | ⑥ Use folk remedies to enhance immunity |
| ⑦ Visit a health facility | ⑧ Others (_________________________) |

1. Do you think MERS is preventable?

① Yes ② No

**III. Questions on experience and perceptions on CAM use**

- Complementary and alternative medicine (CAM) refers to medical products and practices that are not part of conventional medical care. CAM includes traditional Korean medicine, mind-body interventions, biologically-based treatments, manipulative and body-based methods, and energy therapies.

1. Did you ever use any CAM in the past 12 months before the MERS outbreak?

① Yes ② No

1. If you have ever used any CAM ***during the MERS outbreak***, which type of CAM have you used? (“√” all that apply)

| ① Vitamin | ② Probiotics |
| --- | --- |
| ③ Nutritional supplements (_________) | ④ Ginseng/Red ginseng |
| ⑤ Cinnamon | ⑥ *Dioscorea japonica* |
| ⑦ Whole grain/ brown rice/ black beans | ⑧ Garlic/Ginger |
| ⑨ Propolis | ⑩ Acupuncture/ moxibustion/ cupping |
| ⑪ Pray/ meditation | ⑫ Chiropractic |
| ⑬ Physical exercise | ⑭ Massage |
| ⑮ Herbal medicine | ⑯ Green vegetable juice |
| ⑰ Yoga/Aerobics | ⑱ Others (______________) |
| ⑲ Did not use any CAM |  |

1. If you used CAM during the MERS outbreak, what was the purpose of using CAM? (“√” all that apply)

| ① To improve the immune system |
| --- |
| ② To assist in the treatment of disease(s) |
| ③ To reduce pain |
| ④ To aid in psychological comfort |
| ⑤ To prevent disease and maintain/promote health |
| ⑥ To minimize the adverse effects of conventional medicine |
| ⑦ Others (____________________) |

1. Where did you obtain the information on CAM? (“√” all that apply)

| ① Family or relatives | ② Friends or peers |
| --- | --- |
| ③ Mass media (newspaper, radio, TV) | ④ Internet |
| ⑤ Book or magazine | ⑥ Pharmacist |
| ⑦ Others (_________________) |  |

1. Do you intend to recommend CAM modalities to others?

① Yes ② No

1. If you did not use any CAM during the MERS outbreak, what was the reason?

| ① I do not believe that CAM is relevant to disease treatment |
| --- |
| ② I do not trust in the effectiveness of CAM |
| ③ I am worried about the adverse effects of CAM |
| ④ I do not know about CAM |
| ⑤ Others (________________________) |

1. Which health facilities do you usually visit to obtain healthcare services?

| ① Pharmacy | ② Health center (including health posts) |
| --- | --- |
| ③ Traditional Korean medicine hospitals | ④ Local hospitals/ clinics |
| ⑤ Integrative medicine hospitals | ⑥ General/ tertiary hospitals |
| ⑦ Others (_________________) |  |

**IV. Sociodemographic characteristics of study participants**

1. What is your year of birth and age (in years)? Year of birth: 19 __ __ ; age: _________
2. What is your gender? ①Male ② Female
3. What is your marital status? ①Single ② Married ③ Others

30. What is the highest level of education you have completed?

| ① No education (illiterate) | ② Elementary school |
| --- | --- |
| ③ Middle school | ④ High school |
| ⑤ College/ university level | ⑥ Graduate school or above |

31. What is your occupation?

| ① Professional | ② Office worker, government official |
| --- | --- |
| ③ Self-employed | ④ Service sector |
| ⑤ Farmer/fisherman | ⑥ Housewife |
| ⑦ Manual worker | ⑧ Not employed |
| ⑨ Student | ⑩ Others (_________________) |

32. What is the monthly household income (in total)?

| ① < 2 million KRW | ② 2 million – 3.99 million KRW | |  |  |
| --- | --- | --- | --- | --- |
| ③ 4 million – 5.99 million KRW | ④ 6 – 7.99 million KRW | |  |  |
| ⑤ 8 million – 9.99 million KRW | ⑥ ≥ 10 million KRW | |  |  |
|  | | |  | |
|  | | |  | |

33. What is your religion?

| ① None | ② Christianity |
| --- | --- |
| ③ Buddhism | ④ Catholic |
| ⑤ Won-Buddhism | ⑥ Others (_______________) |

34. Where do you live?

| ① Seoul City | ② Gyeonggi, Incheon (metropolitan area) |
| --- | --- |
| ③ Rural district | ④ Provincial city |
| ⑤ Others (_______________) |  |

35. What is the type of housing you live in?

| ① Apartment | ② House (detached house) |
| --- | --- |
| ③ Villa/ multiplex housing | ④ Dormitory |
| ⑤ Others (_______________) |  |

---------------------------------------------Thank you-------------------------------------------------
